# Comparing performance of modern genotype imputation methods in different ethnicities

**DOI:** 10.1038/srep34386

**Published:** 2016-10-04

**Authors:** Nab Raj Roshyara, Katrin Horn, Holger Kirsten, Peter Ahnert, Markus Scholz

**Affiliations:** 1 Institute for Medical Informatics, Statistics and Epidemiology, University of Leipzig, Haertelstrasse 16-18, 04107 Leipzig, Germany; 2 LIFE Center (Leipzig Interdisciplinary Research Cluster of Genetic Factors, Phenotypes and Environment), University of Leipzig, Philipp-Rosenthal Strasse 27, 04103 Leipzig, Germany; 3 Department for Cell Therapy, Fraunhofer Institute for Cell Therapy and Immunology, Perlickstraße 1, 04103 Leipzig, Germany

## Abstract

A variety of modern software packages are available for genotype imputation relying on advanced concepts such as pre-phasing of the target dataset or utilization of admixed reference panels. In this study, we performed a comprehensive evaluation of the accuracy of modern imputation methods on the basis of the publicly available POPRES samples. Good quality genotypes were masked and re-imputed by different imputation frameworks: namely MaCH, IMPUTE2, MaCH-Minimac, SHAPEIT-IMPUTE2 and MaCH-Admix. Results were compared to evaluate the relative merit of pre-phasing and the usage of admixed references. We showed that the pre-phasing framework SHAPEIT-IMPUTE2 can overestimate the certainty of genotype distributions resulting in the lowest percentage of correctly imputed genotypes in our case. MaCH-Minimac performed better than SHAPEIT-IMPUTE2. Pre-phasing always reduced imputation accuracy. IMPUTE2 and MaCH-Admix, both relying on admixed-reference panels, showed comparable results. MaCH showed superior results if well-matched references were available (Nei’s *G*_*ST*_ ≤ 0.010). For small to medium datasets, frameworks using genetically closest reference panel are recommended if the genetic distance between target and reference data set is small. Our results are valid for small to medium data sets. As shown on a larger data set of population based German samples, the disadvantage of pre-phasing decreases for larger sample sizes.

Genotype imputation is now common practice in Genome wide association (GWA) analysis^1^,^2^. Imputation facilitates meta-analyses of studies genotyped at different platforms^3^,^4^,^5^ and is supposed to increase the power of GWA analyses^6^. It is also used for fine mapping efforts^7^. Moreover, genome-wide DNA sequencing is still cost-intensive. Sequencing a part of the population and imputing the other individuals using the sequenced samples as reference is therefore a recommended strategy^8^.

Different reference panels of densely genotyped individuals are available and are used as templates of the haplotype structure for the target data sets^9^,^10^,^11^,^12^,^13^,^14^. For example, HapMap provides publicly available reference panels containing individuals with ancestry from West Africa, East Asia and Europe^10^,^11^. The latest generation of the HapMap reference panel^10^ is known as “HapMap3” and includes about 1.6 million common single nucleotide polymorphisms (SNPs) in 1,184 reference individuals from 11 populations. Thereby, ten 100-kilobase regions in a subset of these individuals were sequenced. Another relevant reference panel is phase3 of the 1000Genomes project^9^,^13^. This dataset comprises a haplotype map of 80 million single nucleotide polymorphisms from 2,504 individuals derived from 27 populations. These reference panels are continuously improved both in sample size, density and quality.

Although genotype imputation is a well-established technique, algorithms and methodological processes are continuously refined. To deal with large reference panels, new imputation frameworks and methods were developed for faster computation. Among these, imputation with pre-phasing of the target dataset is the most popular method currently in use. This strategy is implemented in the frameworks MaCH^15^ plus Minimac (MaCH-Minimac) and IMPUTE2^7^ plus SHAPEIT^16^ (SHAPEIT-IMPUTE2)^17^. Research on imputation relying on pre-phasing strategies claimed that this method results in comparable accuracy compared to no pre-phasing^17^. In the present paper, we aim at verifying this claim by comparing its performance with MaCH-Minimac using the POPRES dataset. Moreover, these two frameworks were further compared with those not relying on pre-phasing, namely MaCH, MaCH-Admix^18^, and IMPUTE2.

Another issue during imputation is how to deal with the continuously increasing amount of mixed ethnicities in large epidemiologic studies. This has raised the question to what extend genotype imputation accuracy may be affected by reference panels which do not exactly match with the ancestry of the target populations. To address this issue, imputation algorithms were further refined so that they can adopt reference panels with individuals from multiple populations. This is done by letting the software choose a “custom” reference panel either in a piecewise manner or for the whole genome. Utilizing recent releases of reference panels, different approaches for the selection of appropriate combined reference panels are discussed: Creating a cosmopolitan reference panel by selecting haplotypes from all of the available reference populations^19^,^20^,^21^,^22^, constructing a reference panel by weighted combination strategies^23^,^24^, by principal component clustering^25^, or by selection based on identity-by-state(IBS)^18^,^20^.

Several software packages are designed to deal with admixed populations. Here, we consider three of the most popular methods: IMPUTE2, SHAPEIT-IMPUTE2 and MaCH-Admix. All these three programs implement an IBS-based strategy for selecting an appropriate reference panel. In contrast to IMPUTE2 or SHAPEIT-IMPUTE2, this is done in a piecewise manner by MaCH-Admix. We compare these three programs with the software frameworks requiring homogeneous populations as reference panel: MaCH and MaCH-Minimac. In summary, we compare a total of five imputation frameworks to assess, how pre-phasing and usage of admixed reference panels affect imputation accuracy in a variety of populations (POPRES^26^). An extensive simulation study was performed for this purpose.

Since sample size of the POPRES panel is small, we studied the dependence of our comparisons on sample size in a larger data set of a population based study of Germany.

## Materials and Methods

### Datasets

We considered subsamples of different ethnic origins taken from a large set of Population Reference Samples (POPRES)^26^. We obtained the POPRES dataset from dbGaP^27^ through dbGaP accession number phs000145.v4.p2. Genome-wide genotyping of these individuals was performed on the Affymetrix (Mountain View, CA) GeneChip 500K Array set with the published protocol for 96-well-plate format. For our simulations study, we considered data of chromosome 22 consisting of 5,637 SNPs. As target sets for imputation, we selected a total of 20 populations for which at least 40 individuals were available. If more than 40 individuals were available, a random subset of N = 40 was selected. Among these populations, 15 were of Caucasian origin: Australian, Canadians, German, French, Swiss-French, Swiss-German, Swiss, Italian, Spanish, Irish, British, Belgian, Portuguese, individuals from former Yugoslavia, a mixed group of east European origin (i.e. a mixture of people from Czech-republic, Hungary, Poland); two populations of South-Asian origin: Indians and Punjabis, one east-Asian population: Japanese, one Mexican population: Mexican, and finally, a mixed-population of African-Americans (AfAm). Since the POPRES subsets contained only small numbers of individuals, we also considered a larger German data set of 2,500 individuals of the LIFE-Adult study, a population-based study carried out in the city of Leipzig. Study design is described elsewhere^28^.

### Quality Control and Masking of SNPs

The original POPRES data was based on the Genomic assembly Affymetrix release 25 NSP25 and STY25 with dbSNP Build 126, released on May 2006. However, the reference panel HapMap3 contains rsIDs and corresponding Affymetrix IDs are annotated with dbSNP build 128. Therefore, it was necessary to match the annotation of the variant names and strand orientation. Strand-matching was performed using “fcGENE”^29^. SNPs with ambiguous strand information were removed. 1,014 SNPs could not be matched and were excluded resulting in a total of 4,623 SNPs eligible for analysis.

The major idea of our simulation study is to define high quality (HQ) SNPs assumed to express true genotypes. These SNPs will then be masked, re-imputed and compared with the original genotypes to assess imputation accuracy. We aimed at masking a reasonable number of HQ SNPs for which imputation quality can be assessed without thinning out the linkage disequilibrium structure too much. Moreover, we prefer to mask common variants which are more informative regarding comparisons of true and imputed genotypes. Therefore, we applied the following SNP filter in order to define HQ SNPs: call rate (CR ≥ 95%), minor allele frequency (MAF ≥ 0.1) and p-values of Hardy Weinberg Equilibrium Test p(HWE) ≥ 0.01. For the latter, we applied an exact stratified test of HWE calculated over all POPRES populations considered^30^. Overall 457 SNPs passed these quality criteria in all data subsets.

Imputation quality of a SNP depends on the number of missing SNP (denoted as missingness here). To assess the impact of the degree of missingness, different percentages of HQ SNPs were masked, namely 50%, 70% or all. To ensure comparability, SNPs masked in the scenario of 50% missingness are also masked in the scenario of 70% missingness and so on.

To study the effect of sample size, we considered 2,500 samples from LIFE-Adult. Genotyping was performed using the Affymetrix Axiom CEU array. Affymetrix power tools with standard settings were used for primary SNP calling. Samples were filtered by the following criteria: dish QC < 0.82, call rate < 0.97, sex mismatch, implausible relatedness issues and PCA outliers (6 SD). SNPs were filtered by the following criteria: call rate < 0.97, Affymetrix cluster measures as recommended (FLD, HetSO and HomRO), number of minor allele < 3, deviation from Hardy-Weinberg equilibrium (p < 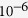), plate association (p < 10^−7^) and minor allele frequency.

For the analysis, we considered 2,474 SNPs in a 10 mega bases area of chromosome 22. HQ-SNPs are defined by MAF > = 0.2, p-value of exact Hardy-Weinberg test > = 0.5, call rate > = 0.995. A total of 522 SNPs fulfilled these criteria and were masked and re-imputed accordingly. To study the impact of sample size, we considered randomly chosen subsets of the original data set of sizes 2500, 1000, 500, 250, 100 and 40. Here, the larger data set always contains the smaller one.

### Reference Panel

In HapMap project, genotyping was performed directly, while the 1000 Genomes dataset relies (at least partly) on low depth whole genome sequencing data. Therefore, HapMap has still the higher accuracy and was chosen as reference panel for the present study^10^,^11^. Here, we used the pre-formatted HapMap3 reference panel. Imputation with MaCH and MaCH-Minimac were performed using the reference panels that were best matched with the ancestry of the target population. This strategy was considered as standard to compare its results with those of MaCH-Admix, IMPUTE2 and SHAPEIT-IMPUTE2, the frameworks which adopt admixed reference panels. Appropriate reference panels: CEU, YRI, MEX and JPT + CHB provided by MaCH software developers through their homepage^31^ were used for imputing the target data sets. The best matched reference was selected by minimizing the genetic similarity measure Nei’s *G*_*ST*_ between the target populations and available reference panels as recommended elsewhere^32^.

IMPUTE2 uses a mixed cosmopolitan reference panel collected from a variety of sampling locations in Africa, Asia, Europe and America. It automatically selects a ‘custom’ reference panel separately for each individual during imputation. We downloaded the mixed reference panel created from the samples of the HapMap3 project available at the IMPUTE2 website^33^ and used it for our purposes. This mixed reference panel consists of haplotypes of a total of 1,011 individuals genotyped on 20,084 SNPs at chromosome 22. Since our aim is to compare IMPUTE2 and MaCH-Admix, we used the same mixed reference panel by converting the reference of IMPUTE2 to MaCH-Admix format using fcGENE^29^.

Due to the fact that the overlap of HapMap3 and Axiom CEU array was rather small, we decided to impute our LIFE-Adult samples with 1000 Genomes reference (Phase 1 Release V3)^34^,^35^.

### Imputation

Imputation was performed separately for each data subset using five different imputation frameworks with or without pre-phasing or usage of admixed reference panels. Table 1 compares the frameworks regarding these options.

For imputation with MaCH, version 1.0.18.c, we first estimated imputation error rate and recombination rate in the haplotype panels by running the “greedy” algorithm for 30 iterations. These two model parameters were then used to determine the posterior probabilities of each genotype in the second step^15^. MaCH calculates the software specific measure “Rsq” to assess imputation quality^15^.

To perform imputation with MaCH-Minimac, we first determined the haplotypes of target data sets using MaCH software. Then the pre-phased data were imputed with Minimac, version Minimac2 from 2014.9.15.

For imputation with IMPUTE2, version 2.3.1 was used with default parameters. We performed imputation by splitting chromosome 22 in 6 chunks of equal size 5.711 MB as recommended^33^. This can be done by providing the lower and upper boundaries of base pair position with IMPUTE2 command option “-int”. Format conversion and IMPUTE commands including the lower and upper boundaries of each chunk were generated by fcGENE^29^. The population-genetic model used by IMPUTE2 requires an effective population size as input parameter. Although different human populations have different effective sizes, IMPUTE software providers recommend a large value of about 20000 for the parameter “−Ne” as universal value through which they achieved high accuracy across all population groups. To avoid margin effects while chunking genotypic region, IMPUTE2 uses an internal buffer region (default is 250 kb) on either side of the analysis interval^33^. Imputation processes were run in a parallel way to speed up the computational runtime. At the end of each computation, we extracted the imputation quality scores. As suggested by the software providers^33^, the best strategy for imputing genotype data with IMPUTE2 is first to phase the study population with SHAPEIT^16^,^36^ and then impute the phased data with IMPUTE2. We followed this strategy denoted as “SHAPEIT-IMPUTE2” (using SHAPEIT version v2 r790) in the following.

For imputation with MaCH-Admix^18^, version v2.0.203, we used the integrated default run mode where model parameters like recombination rate and error rates are automatically determined before calculating genotypes and imputation quality. Admixed reference panels used for MaCH-Admix were created from corresponding IMPUTE2-formatted reference panels which were downloaded from the home page of IMPUTE2^33^. We also used the implemented two step method of MaCH-Admix which is similar to those of MaCH. Results were similar to those of the default strategy (not shown). All software-specific commands are provided in the supplement material S1.

### Measures of imputation accuracy

Direct comparison of true and imputed genotypes: Although, imputation software usually provide measures of imputation accuracy, these measures typically are software specific, hampering comparisons across software. To circumvent this issue, we masked good quality SNPs and re-imputed them allowing an objective assessment of imputation accuracy. Comparisons of true genotypes and imputed genotype distributions were performed in the following ways: First, we compared the original true genotypes of masked HQ SNPs with corresponding best-guess genotypes. For this type of comparison, we also analysed the posterior probabilities of both, the correctly and incorrectly imputed best-guess genotypes. In another approach, we compared true genotypes with estimated posterior distributions by applying platform independent Hellinger and SEN scores^37^. While the SEN score essentially compares the expectations of genotype distributions, Hellinger score is a measure of the agreement of genotype probabilities. Hellinger score ≥0.45 ensures that the probability of best-guess genotypes is at least 0.49 and the best-guess genotype matches with the original genotypes in almost all cases (see results below). Therefore, this cut-off was used to define well-imputed genotypes in the following.

To find out whether there are significant differences between the imputation scenarios, we formally compared percentages of well-imputed genotypes by McNemar’s test or raw quality measures by Wilcoxon signed rank test. Analyses were performed with the statistical software package R (www.r-project.org). We used 5% as significance threshold throughout all analyses, i.e. we refrained from correcting for multiple comparisons. Since we generally compared the best scenario against the others, we performed one-sided tests throughout. For these analyses, masked HQ SNPs were considered as independent in view of the relatively weak linkage structure of this subset. Only 1% of HQ SNP pairs showed a linkage disequilibrium of *r*^*2*^ ≥ 0.1.

Comparisons using software specific scores: Software specific imputation accuracy measures comprise MaCH-Rsq and IMPUTE-info scores. Both are defined on a SNP-wise rather than genotype level. Although these quality scores do not allow comparisons across software, they are often used to remove poorly imputed SNPs in practice. Hence, we consider these scores in a secondary analysis.

Alternatively, one could calculate the correlation between imputed allele dosages and true genotypes separately for each SNP to assess its imputation quality. This measure is also software independent but does not account for random agreement due to the prior distribution of the imputed genotypes. Analysis shows that this measure is in strong agreement with MaCH-Rsq especially for larger sample sizes (Supplementary Figure S3).

## Results

### Characteristics of quality scores for comparing different imputation frameworks

**Figure 1:**
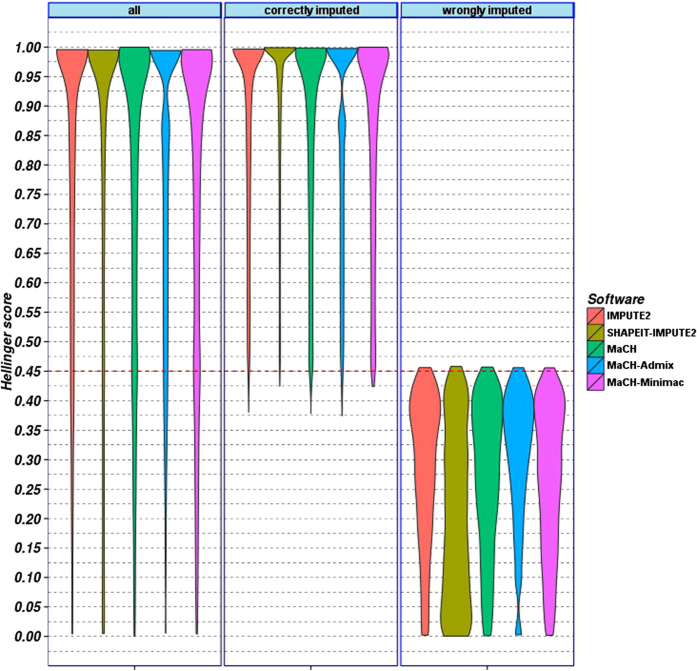
Violin plot of Hellinger scores of genotypes imputed with five different frameworks. Results of African-Americans (AfAm) population are shown. We present results for all imputed genotypes, and separately, for cases where best guess genotypes match true genotypes (correctly imputed) or not (wrongly imputed). A Hellinger score > = 0.45 almost always ensured that the best-guess genotype matches the true genotype.

Initially, we characterized and compared our imputation accuracy scores (Hellinger score, SEN score and percentages of best guess genotypes matching original genotypes) and the software specific scores (MaCH-Rsq and IMPUTE-info). First, we aimed at identifying a cut-off for Hellinger score to distinguish between correctly imputed genotypes (CIGs) and wrongly imputed genotypes (WIGs). Our analysis revealed that genotype distributions with Hellinger score > = 0.45 always had posterior probability of best-guess genotypes greater than 0.49 and this was sufficient to match the original genotype in almost all cases (see Fig. 1 for AfAm population, representation as boxplots can be found as Supplementary Figure S1). This applies for all POPRES populations considered.

**Figure 2:**
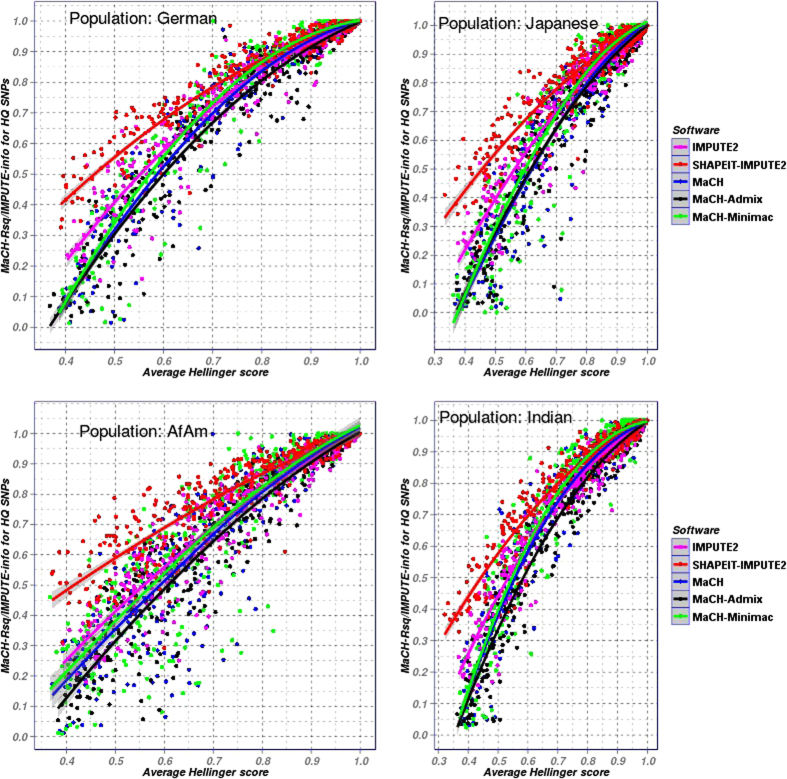
Scatterplot between average Hellinger score and Mach-Rsq/IMPUTE-info score for four different POPRES populations imputed with MaCH (using YRI reference panel), MaCH-Minimac (using YRI reference panel), MaCH-Admix, IMPUTE2 and SHAPEIT-IMPUTE2 (using admixed reference panels). For the same Hellinger score, Info scores of SHAPEIT-IMPUTE2 are clearly inflated compared to IMPUTE2.

Since most of the research work comparing software performance^17^,^20^ are based on the software specific measures (MaCH-Rsq and IMPUTE-info), we studied these measures in relation to the Hellinger score. Figure 2 shows the results of four example populations of POPRES (German, AfAm, Indian and Japanese). Here, MaCH-Rsq and IMPUTE-info score are only roughly correlated with Hellinger score. Interestingly, for a given value of Hellinger score, SHAPEIT-IMPUTE2 showed clearly higher info scores compared to IMPUTE2. Since Hellinger score is an objective measure of imputation accuracy, we conclude that the info measures of SHAPEIT-IMPUTE2 are inflated. The same trend was observed for MaCH-Minimac versus MaCH but with much lesser magnitude.

Of note, MaCH-Rsq and IMPUTE-info strongly depend on the underlying reference panel and can predict the imputation accuracy only under the assumption that the underlying reference panel is genetically very close to the target data set^32^. In contrast, Hellinger score is independent of software and makes no assumptions regarding the underlying reference panel. Therefore we decided to consider Hellinger score as the primary measure for imputation accuracy in this analysis.

**Figure 3:**
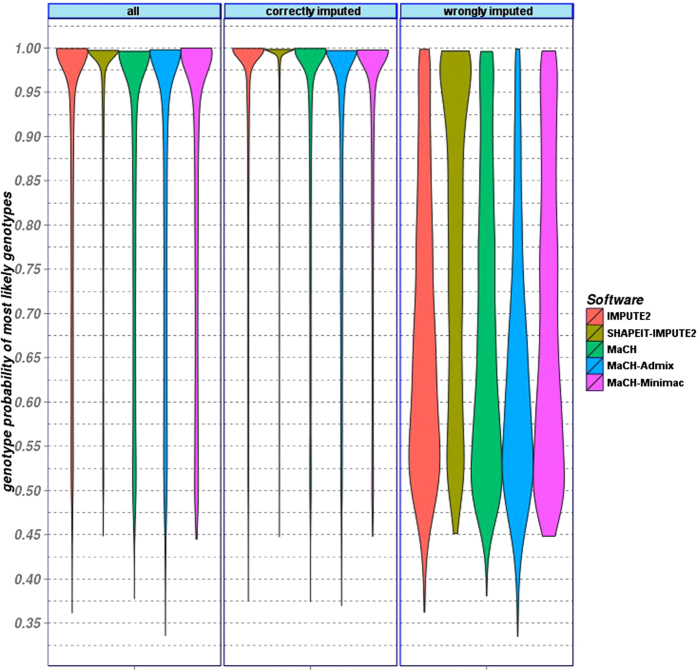
Violin plot of posterior probabilities of best guess genotypes in AfAm population. All imputation frameworks were used with default parameters and reference panels. SHAPEIT-IMPUTE2 shows considerably higher posterior probabilities for wrongly imputed SNPs.

To study inflated accuracy scores for SHAPEIT-IMPUTE2 shown in Fig. 2 in more detail, we analyzed the probability of best-guess genotypes for each of the five frameworks. Results are shown in Fig. 3 (see also Supplementary Figure S2 for alternative representation as box-plots). Interestingly, while the distribution of posterior probabilities of best-guess genotypes are similar for correctly imputed genotypes (CIGs), the distribution of the SHAPEIT-IMPUTE2 values is different for wrongly imputed genotypes (WIGs). In contrast to the other frameworks, SHAPEIT-IMPUTE2 apparently estimates high posterior probabilities also for WIGs. In the sense of Fig. 3, MaCH-Admix shows the most desirable behavior, i.e. low probabilities for wrong best-guess genotypes.

### Comparison of Frameworks using Admixed Reference Panels vs Best Matched Reference Panels

Next, we aimed at answering the question if and under which circumstances is the usage of admixed reference panels advantageous compared to specific references panels matched to the target population. More precisely, we analysed the impact of genetic similarity between reference and target population on imputation accuracy. For the imputation frameworks relying on a specific reference, we selected the reference with smallest value of Nei’s 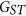 as explained in the methods section. We used percentage of Hellinger score > = 45% as primary quality score. A total of 20 populations were analysed with all five imputation frameworks considered (Table 2).

When considering frameworks without pre-phasing (MaCH, MaCH-Admix, IMPUTE2), we found that usage of admixed reference panels (MaCH-Admix, IMPUTE2) was advantageous only if the genetic difference between target and reference population was large. In more detail, performance was better when Nei’s *G*_*ST*_ was close to or greater than about 0.01 which is the case in 6 of the 20 POPRES samples. For POPRES population AfAm, for which no well-matched reference is available, MaCH is clearly outperformed by MaCH-Admix and IMPUTE2. In other words, for well-matched references and homogenous populations as in most of our POPRES samples, the usage of specific references results in superior imputation quality. Considering the pre-phasing frameworks (MaCH-Minimac and SHAPEIT-IMPUTE2) we found that both are clearly outperformed by their counterparts not relying on pre-phasing (MaCH and IMPUTE2, respectively). Results were similar when considering other measures of imputation quality like SEN score, percentages of correctly imputed genotypes based on best guess genotype, and software specific measures of imputation accuracy (supplementary Table S1, S2, and S3, respectively).

We observed a general trend of lower imputation qualities for larger genetic distances to the best matching reference. This also applies for imputation frameworks relying on mixed references (see Supplementary Figure S4).

### Comparison of Frameworks Using Admixed Reference Panels

Table 3 shows the results of the comparison of imputation frameworks relying on admixed reference panels (MaCH-Admix, IMPUTE2 and SHAPEIT-IMPUTE2). For this purpose, we also consider three different missing scenarios to account for the impact of missingness on efficacy of the imputation frameworks. Again, we used McNemar’s test to compare the scenarios.

MaCH-Admix and IMPUTE2 showed comparable performance. IMPUTE2 had an advantage compared to MaCH-Admix especially for larger percentages of missingness but the difference was insignificant in general. In contrast, SHAPEIT-IMPUTE2 always showed significantly inferior results.

Results for SEN score are similar (results not shown). We also determined the percentage of correctly imputed best-guess genotypes (Table 4). Results are similar to those of the Hellinger score except for the fact that here, one can observe a slight but insignificant advantage of MaCH-Admix compared to IMPUTE2. Hence, IMPUTE2 tends to be more confident at certain SNPs while MaCH-Admix has a slightly higher average yield of correctly guessed genotypes. Again, SHAPEIT-IMPUTE2 showed significantly poorer performance than the other frameworks.

### Comparison of frameworks relying on pre-phasing

Table 5 shows results of the comparison of frameworks using pre-phasing (MaCH-Minimac and SHAPEIT-IMPUTE2). As primary quality measure, percentage of genotypes with good Hellinger score (≥0.45) was used. As observed in Table 2, small Nei’s *G*_*ST*_ between reference and target population were advantageous for MaCH-Minimac relying on specific reference panels. However, there was a trend that the difference to SHAPEIT-IMPUTE2 became smaller when missingness increases. For those populations, whose genetic distances from the best-matching reference population is large, SHAPEIT-IMPUTE2 performed slightly better than MaCH-Minimac, however in many cases the difference was insignificant.

Similar results are obtained for the SEN score (see Supplementary Table S4). Again, we analysed the percentage of correctly guessed genotypes (Table 6). We found that MaCH-Minimac performed always better than SHAPEIT-IMPUTE2 except in the case of 70% and 100% missing scenarios for “AfAm” population. In these two scenarios, SHAPEIT-IMPUTE2 showed insignificantly better performance. This underlines the importance of admixed references for imputation of AfAm for which no well matching reference is available.

### Impact of sample size

The impact of sample size on the performance of imputation frameworks was studied in LIFE-Adult. Results are shown in Table 7. Again, methods without pre-phasing have higher accuracy than their counterparts relying on pre-phasing. But the difference becomes smaller with increasing sample size. MaCH is superior to IMPUTE2 for small datasets but for larger datasets, the opposite is true.

## Discussion

In the present paper, we compared the imputation frameworks MaCH, IMPUTE2, MaCH-Admix, MaCH-Minimac and SHAPEIT-IMPUTE2 in a comprehensive simulation study of POPRES samples. We were interested if and under which circumstances pre-phasing or usage of admixed references panels is advantageous.

Genotype imputation is nowadays common in genome-wide data analysis. Although, frameworks such as MaCH, IMPUTE2 and Beagle are well established and result in generally good imputation quality, there are several attempts regarding further improvements. First, in order to deal with larger data sets, pre-phasing was established which significantly accelerates imputation speed^17^. According to this strategy, the haplotypes underlying the target dataset are estimated first. Then, these haplotypes were used to estimate the genotypes. The two imputation frameworks SHAPEIT-IMPUTE2 and MaCH-Minimac adopt this concept^17^. While SHAPEIT-IMPUTE2 uses an admixed reference panel as input and let the software choose a “custom” reference panel, MaCH-Minimac basically depends on a reference panel that is best matched with the target dataset. Second, admixed populations becoming more and more frequent in genetic epidemiologic research. Therefore, frameworks accepting admixed reference populations were developed^18^,^20^. There is also some hope that admixed references might improve the imputation accuracy for populations for which no well-matching reference is at hand. The software IMPUTE2 and MaCH-Admix implemented this approach. Both software implemented an IBS-based strategy for selecting the reference panel but the latter’s IBS-matching strategy is in a piecewise manner. So far, only few published studies compared the relative performance of imputation concepts of pre-phasing or accounting for admixture^17^. Conclusions from these studies are limited since their findings were based on the IMPUTE-Info score as quality measure, only. According to our results (Fig. 2), IMPUTE-Info score strongly depends on the reference panel used. In our study, we used scores that allow a direct comparison of imputed and true genotypes. Using these measures, we compared the above mentioned imputation frameworks in a comprehensive simulation study.

Our simulation study is based on the general idea of masking SNPs, re-imputing them and comparing the results using a variety of measures. Only good quality SNPs were masked to ensure that expressed genotypes are correct with high certainty. As in earlier studies^37^, we considered Hellinger score as the primary outcome of the comparison of masked and re-imputed genotypes. The score is maximal if and only if the two genotype distributions coincide. In our simulation study, we showed that a Hellinger score > = 0.45 almost ensures that the best-guess genotype is correct. This applies for all software and simulation scenarios considered. We studied SEN score and percentage of correct best-guess genotypes as alternative objective measures of imputation quality. Results were in general similar to those of Hellinger score.

Although, software specific measures of imputation quality such as MaCH-Rsq and IMPUTE-info are widely used to assess imputation accuracy, our results suggest that these measures should not be used as objective (absolute) measures of imputation accuracy. First, these measures depend on the reference panel considered^32^. Second, we observed a strong inflation of IMPUTE-info for the framework SHAPEIT-IMPUTE2 and numerous best-guess genotypes are wrong even if IMPUTE-info is high. This could explain for example the results of Howie *et al*.^17^ which was based on IMPUTE-info scores. This study concluded that SHAPEIT-IMPUTE2 and IMPUTE2 perform similarly. However, our simulation study shows that IMPUTE2 without pre-phasing is considerably better. Moreover, we recommend applying higher IMPUTE-info thresholds for SHAPEIT-IMPUTE2 than for IMPUTE2 to achieve similar imputation quality. We generally observed that software frameworks with pre-phasing strategy performed inferior compared to their equivalents without pre-phasing. Thus, there is a trade-off between imputation accuracy and cost of computational time. However, our analysis of LIFE-Adult shows that the disadvantage of pre-phasing decreases for larger sample sizes.

Regarding the performance of admixed reference panels, it was necessary to study a variety of genetic ethnicities. Therefore, we created 20 different ethnic data subsets of chromosome 22 from the POPRES project^26^. Each ethnic data subset consisted of equal numbers of individuals (N = 40). Limitations of this approach are the relatively low number of cases as well as the fact that no true admixed target population was considered. Therefore, results might be valid only for small or medium-sized data sets.

As imputation references, we considered the HapMap3 samples CEU, YRI, MEX and JPT + CHB as possible best-matched references. For our POPRES samples, we selected the reference with minimal Nei’s *G*_*ST*_ as recommended^32^. For software relying on admixed references, a corresponding HapMap reference was selected. Usage of HapMap references is a limitation of our study. However, in view of the small case numbers of POPRES populations, imputation of rare and low frequency variants is futile (see also supplementary figure S5), and therefore, we have to focus on common variants which are well represented in the HapMap panels.

Comparison of MaCH-Admix using an admixed reference versus MaCH using a specific reference showed that the specific references are advantageous as long as there is a well-matching reference population. A cut-off of Nei’s *G*_*ST*_ of 0.01 could serve as a rough decision rule whether an admixed reference should be preferred. The software relying on admixed references without pre-phasing, MaCH-Admix and IMPUTE2, performed similarly. However, one has to acknowledge here that this was shown only for small genetically homogeneous populations as those of POPRES.

In summary, admixed references outperformed best-matched references only if the genetic distance was large (Nei’s *G*_*ST*_ > 0.01). Pre-phasing reduces imputation accuracy, but the difference becomes smaller for larger data sets. Relative measures of imputation accuracy such as MaCH-Rsq and IMPUTE-info should be considered with caution when interpreting and comparing imputation accuracy, since they depend on the reference and the imputation framework. Our conclusions are valid for genetically homogenous populations of small to moderate sample size.

## Additional Information

**How to cite this article**: Roshyara, N. R. *et al*. Comparing performance of modern genotype imputation methods in different ethnicities. *Sci. Rep.* **6**, 34386; doi: 10.1038/srep34386 (2016).

## Supplementary Material

Supplementary Information

## Figures and Tables

**Table 1:** Imputation Frameworks analysed: Frameworks differ with respect to usage of pre-phasing or admixed versus specific reference panels.

Imputation software/framework	Pre-phasing	Use of admixed reference panel
MaCH-Minimac	Yes	No
SHAPEIT-IMPUTE2	Yes	Yes
Mach-Admix	No	Yes
MaCH	No	No
IMPUTE2	No	Yes

We aim at comparing the impact of these features on imputation accuracy.

**Table 2:** Comparison of percentages of genotypes with good Hellinger scores (> = 0.45) obtained for 20 different POPRES samples with either MaCH, MaCH-Minimac, MaCH-Admix, IMPUTE2, or SHAPEIT-IMPUTE2.

Population	MaCH and MaCH-Minimac framework(Best-matched Reference Panel)				Mixed Reference Panel		
	Reference Panel	Nei’s *G*_*ST*_	MaCH	MaCH-Minimac	MaCH-Admix	IMPUTE2	SHAPEIT-IMPUTE2
Australian	CEU	0.0078287	***89.690***	88.334*	89.031*	89.393	88.081*
British	CEU	0.0078541	***90.779***	89.189*	89.973*	90.231*	88.547*
Canadian	CEU	0.0078631	***90.218***	88.583*	89.603*	89.702*	87.985*
Swiss.French	CEU	0.0079978	***89.761***	88.495*	89.098*	89.153*	87.864*
French	CEU	0.0080226	***90.085***	88.277*	89.241*	89.291*	88.255*
German	CEU	0.0080485	***90.240***	88.81*	89.478*	89.703*	88.338*
Irish	CEU	0.0081449	***90.286***	89.155*	89.49*	89.704*	88.541*
Swiss	CEU	0.0082549	***89.774***	88.151*	89.264*	89.357*	87.937*
Belgians	CEU	0.0084603	***90.273***	89.062*	89.992	90.009	88.291*
Swiss.German	CEU	0.0086417	***89.706***	88.456*	89.366*	89.081*	87.848*
eastEU	CEU	0.0088483	***89.500***	88.256*	88.991*	89.144	87.851*
Portuguese	CEU	0.0096742	88.569	87.34*	88.410	***88.613***	87.675*
Spanish	CEU	0.0096786	***89.220***	87.909*	89.023	88.985	87.706*
Italian	CEU	0.0105699	***88.934***	88.025*	88.781	88.742	87.28*
From Yugoslavia	CEU	0.0108079	***89.049***	87.832*	88.643*	88.819	87.629*
Mexican	MEX	0.0108799	89.137*	87.908*	89.477*	*90.059*	88.188*
AfAm	YRI	0.0188273	82.603*	80.86*	***86.211***	86.123	83.437*
Punjabi	CEU	0.0244462	86.767*	86.257*	87.951	***88.137***	87.14*
Indian	CEU	0.0247062	86.441*	85.202*	87.527	***87.845***	86.315*
Japanese	CHB.JPT	0.0330444	89.089*	88.391*	89.524*	***90.132***	88.575*

For Imputation with MaCH and MaCH-Minimac framework, the best matched reference panels based on Nei’s *G*_*ST*_ were selected. Nei’s *G*_*ST*_ values and corresponding reference panels are also presented. Imputation frameworks with best results are marked with bold italic letter for each population and those scenarios which are significantly different from the best scenario are marked with an asterisk. McNemar’s test was used to determine significant differences of alternative scenarios to the best scenario.

**Table 3:** Percentage of Genotypes with good Hellinger score (> = 0.45) for three imputation frameworks considering mixed reference panels:

Country	MaCH-Admix			IMPUTE2			SHAPEIT-IMPUTE2		
Missing percentage	50%	70%	100%	50%	70%	100%	50%	70%	100%
German	***91.28***	90.26	90.06	91.1	***90.37***	***90.07***	89.2*	89.17*	88.88*
Swiss-German	90.37	89.37	***89.63***	***91.08***	***90.02***	89.4	88.19*	88.29*	88.28*
Belgians	91.34	90.5	***90.34***	***91.75***	***90.75***	90.23	89*	88.83*	88.63*
Spanish	***90.36***	89.7	89.29	90.35	***89.77***	***89.52***	88.19*	88.00*	88.06*
French	90.75	89.67	89.32	***90.84***	***89.9***	***89.59***	88.85*	88.59*	88.89*
Irish	90.84	90.07	89.62	***91.19***	***90.3***	***89.93***	88.64*	88.59*	88.92*
Italian	***90.57***	***89.94***	***89.56***	90.46	89.57	89.56	87.93*	88.03*	87.94*
Portuguese	***90.29***	89.15	88.61	90.23	***89.35***	***89.13***	87.84*	87.78*	87.99*
Swiss-French	90.77	89.76	89.39	***91.13***	***90.1***	***89.79***	89.02*	88.69*	88.71*
Swiss	90.65	89.73*	89.8	***91.01***	***90.53***	***89.87***	88.72*	88.77*	88.64*
British	91.62	90.51	90.61	***91.79***	***90.94***	***90.71***	89.16*	89.35*	89.15*
FromYugoslavia	90.1	89.15	88.87	***90.26***	***89.39***	***89.34***	88.23*	87.83*	88.09*
Canadian	***91.41***	90.23	90.08	91.32	***90.61***	***90.25***	89.08*	88.86*	88.7*
Mexican	91.49	90.64	90.37*	***91.87***	***91.07***	***91.13***	89.54*	89.42*	89.07*
Australian	90.91	89.7*	89.29	***91.12***	***90.29***	***89.8***	88.62*	89.08*	88.68*
Japanese	91.7	90.59*	90.34*	***91.84***	***91.11***	***91.16***	89.84*	89.86*	89.76*
AfAm	***87.89***	***87.34***	86.25	87.85	86.87	***86.34***	83.49*	83.36*	83.8*
Punjabi	***90.14***	89.14	88.91	89.95	***89.43***	***89.1***	87.69*	88.03*	88.04*
Indian	89.61	***88.78***	88.44	***90.09***	88.78	***88.67***	87.37*	87.36*	87.27*
eastEU	***90.8***	89.6	89.27	90.8	***89.8***	***89.52***	88.17*	88.21*	88.17*

20 Popres population were studied. Different percentages of HQ-SNPs were masked (50%, 70%, and 100%) and re-imputed. The best software framework for each population and degree of missingness is presented in bold italic letters. An asterisk (*) indicates whether the other software frameworks perform significantly worse for the corresponding missingness scenario.

**Table 4:** Percentage of most likely genotypes which agree with the original genotypes for three imputation frameworks considering mixed reference panels:

Software->	MaCH-Admix			IMPUTE2			SHAPEIT-IMPUTE2		
**Country**	**50%**	**70%**	**100%**	**50%**	**70%**	**100%**	**50%**	**70%**	**100%**
German	***92.00***	***91.18***	***91.00***	91.35*	90.64*	90.26*	89.03*	89.29*	88.76*
Swiss-German	91.09	***90.27***	***90.44***	***91.10***	90.20	89.8*	88.22*	88.31*	88.33*
Belgians	***91.67***	***91.10***	***90.75***	91.44*	90.47*	90.08*	88.54*	88.44*	88.16*
Spanish	***91.10***	***90.43***	***90.29***	90.56*	89.99*	89.84	87.96*	87.87*	87.98*
French	***91.44***	***90.31***	***90.15***	91.12	90.07	89.84	88.85*	88.62*	88.82*
Irish	***91.51***	***90.74***	***90.62***	91.23	90.43	90.20	88.53*	88.47*	88.71*
Italian	***91.15***	***90.60***	***90.38***	90.80	89.77*	89.97*	87.98*	88.07*	87.98*
Portuguese	***90.81***	***89.95***	***89.60***	90.34	89.55*	89.34	87.63*	87.63*	87.93*
Swiss-French	***91.43***	***90.63***	***90.16***	91.21	90.24*	90.02	88.86*	88.74*	88.73*
Swiss	***91.36***	90.40	***90.47***	91.29	***90.68***	90.13	88.59*	88.7*	88.58*
British	***92.33***	***91.28***	***91.33***	91.92*	91.18	91.03	89.16*	89.24*	89.03*
FromYugoslavia	***90.92***	***90.01***	***89.80***	90.51*	89.64	89.53	88.24*	87.81*	87.98*
Canadian	***91.99***	***91.15***	***91.21***	91.52*	90.94	90.57*	89.07*	88.78*	88.6*
Mexican	91.82	***91.23***	91.02	*91.93*	90.98	***91.21***	89.27*	89.19*	88.84*
Australian	***91.47***	***90.63***	90.01	91.20	90.45	***90.03***	88.41*	88.92*	88.46*
Japanese	***92.17***	***91.23***	90.97	91.7*	91.19	***91.00***	89.52*	89.5*	89.43*
AfAm	***88.80***	***87.89***	***87.16***	88.02*	87.24*	86.79	83.48*	83.42*	83.69*
Punjabi	***90.86***	***90.08***	***89.88***	90.28*	89.73	89.47*	87.62*	88.07*	88.05*
Indian	***90.33***	***89.67***	***89.20***	90.22	89.21	89.14	87.28*	87.29*	87.17*
eastEU	***91.51***	***90.22***	***90.24***	91.10	90.07	89.75*	88.07*	88.17*	88.11*

20 Popres population were studied. Different percentages of HQ-SNPs were masked (50%, 70%, and 100%). The best software framework for each population and degree of missingness is presented in bold italic letters. An asterisk (*) indicates whether the other software frameworks perform significantly worse for the corresponding missingness scenario.

**Table 5:** Percentage of genotypes with good Hellinger score (> = 0.45) for imputation frameworks with pre-phasing strategy:

Country	Genetic similarity		MaCH-Minimac			SHAPEIT-IMPUTE2		
	Reference Panel	Nei’s *G*_*ST*_	50%	70%	100%	50%	70%	100%
Australian	CEU	0.0078287	***90.58***	***89.26***	88.67	88.62*	89.08	***88.68***
British	CEU	0.0078541	***90.95***	***90.02***	***89.88***	89.16*	89.35*	89.15*
Canadian	CEU	0.0078631	***90.74***	***89.34***	***88.88***	89.08*	88.86	88.7
Swiss.French	CEU	0.0079978	***90.13***	***89.03***	***88.95***	89.06*	88.69	88.71
French	CEU	0.0080226	***89.9***	***89.64***	88.56	88.85*	88.59*	***88.89***
German	CEU	0.0080485	***90.9***	***89.72***	***89.37***	89.20*	89.17	88.88
Irish	CEU	0.0081449	***90.43***	***89.54***	***89.38***	88.64*	88.59*	88.92
Swiss	CEU	0.0082549	***90.44***	***89.1***	***88.74***	88.73*	88.77	88.64
Belgians	CEU	0.0084603	***90.9***	***89.84***	***89.54***	88.10*	88.83*	88.64*
Swiss.German	CEU	0.0086417	***90.14***	***88.87***	***88.69***	88.19*	88.29	88.28
eastEU	CEU	0.0088483	***89.93***	***88.83***	***88.59***	88.18*	88.21*	88.17
Portuguese	CEU	0.0096742	***89.33***	***88.29***	**87.77**	87.84*	87.78	***87.99***
Spanish	CEU	0.0096786	***89.51***	***88.45***	***88.26***	88.19*	87.1	88.06
Italian	CEU	0.0105699	***89.47***	***88.63***	***88.62***	87.93*	88.03	87.94*
From Yugoslavia	CEU	0.0108079	***89.86***	***88.58***	***88.51***	88.23*	87.83*	88.09
Mexican	MEX	0.0108799	***90.03***	89.33	88.78	89.54	***89.42***	***89.07***
AfAm	YRI	0.0188273	83.11	81.92*	81.72*	***83.49***	***83.36***	***83.8***
Punjabi	CEU	0.0244462	***87.96***	87.39	86.98*	87.69	***88.03***	***88.04***
Indian	CEU	0.0247062	***87.66***	86.79	86.13*	87.37	***87.36***	***87.27***
Japanese	CHB.JPT	0.0330444	***89.9***	88.88*	89.06	89.71	***89.71***	***89.67***

The rows of the table are arranged with increasing order of genetic distance between target population and best matched reference. Different percentages of HQ-SNPs were masked (50%, 70%, and 100%). The best software framework for each population and degree of missingness is presented in bold italic letters. An asterisk (*) indicates whether the other software framework perform significantly worse for the corresponding scenario. MaCH-Minimac tends to be advantageous for small distances between target and reference population and for lower percentages of missingness.

**Table 6:** Percentage of well-imputed best-guess genotypes for two imputation frameworks relying on pre-phasing.

Country			MaCH-Minimac			SHAPEIT-IMPUTE2		
	Best matched reference	Nei’s *G*_*ST*_	50%	70%	100%	50%	70%	100%
Australian	CEU	0.0078287	***91.29***	***90.41***	***89.82***	88.77*	89.28*	88.82*
British	CEU	0.0078541	***91.58***	***90.98***	***90.7***	89.34*	89.41*	89.20*
Canadian	CEU	0.0078631	***91.44***	***90.28***	***89.93***	89.28*	88.98*	88.81*
Swiss.French	CEU	0.0079978	***90.81***	***90.02***	***89.9***	89.05*	88.93*	88.92*
French	CEU	0.0080226	***90.73***	***90.68***	***89.74***	89.06*	88.83*	89.02*
German	CEU	0.0080485	***91.54***	***90.68***	***90.29***	89.16*	89.43*	88.88*
Irish	CEU	0.0081449	***91.11***	***90.56***	***90.34***	88.83*	88.76*	89.00*
Swiss	CEU	0.0082549	***91.08***	***90.03***	***89.71***	88.83*	88.94*	88.82*
Belgians	CEU	0.0084603	***91.45***	***90.77***	***90.29***	89.14*	89.04*	88.76*
Swiss.German	CEU	0.0086417	***90.72***	***89.63***	***89.47***	88.37*	88.45*	88.48*
eastEU	CEU	0.0088483	***90.63***	***89.76***	***89.49***	88.22*	88.32*	88.27*
Portuguese	CEU	0.0096742	***90.02***	***89.01***	***88.77***	87.88*	87.88*	88.18
Spanish	CEU	0.0096786	***90.24***	***89.4***	***89.15***	88.16*	88.073*	88.18*
Italian	CEU	0.0105699	***90.2***	***89.43***	***89.54***	88.12*	88.19*	88.11*
From Yugoslavia	CEU	0.0108079	***90.36***	***89.43***	***89.41***	88.45*	88.01*	88.18*
Mexican	MEX	0.0108799	***90.72***	***90.2***	***89.72***	89.58*	89.51	89.15
AfAm	YRI	0.0188273	***84.09***	82.79	82.72	83.66	***83.59***	***83.86***
Punjabi	CEU	0.0244462	***88.68***	***88.31***	88.03	87.8	88.25	***88.22***
Indian	CEU	0.0247062	***88.67***	***87.97***	87.24	87.52	87.53	***87.41***
Japanese	CHB.JPT	0.0330444	***90.77***	***90.05***	***90.18***	89.80*	89.76	89.75

The rows of the table are arranged with increasing order of genetic distance between target population and best matched reference measured by Nei’s *G_ST_*. Different percentages of HQ-SNPs were masked (50%, 70%, 100%). The best software framework for each population and degree of missingness is presented in bold italic letter. An asterisk (*) indicates whether the other software framework perform significantly worse for the corresponding scenario.

**Table 7:** Dependence of imputation accuracy on sample size studied in LIFE-Adult.

Reference Panel	MaCH and MaCH-Minimac framework (Best-matched Reference Panel)		Mixed Reference Panel		
	MaCH	MaCH-Minimac	MaCH-Admix	IMPUTE2	SHAPEIT-IMPUTE2
	CEU	CEU	Mixed	Mixed	Mixed
Sample size					
40	***92.35***	90.05*	90.86*	92.23	90.06*
100	***92.38***	91.38*	90.83*	92.27	91.11*
250	***92.39***	91.86*	90.64*	92.27*	91.57*
500	92.29	91.80*	90.30*	***92.33***	91.69*
1000	92.31*	91.86*	90.18*	***92.41***	91.83*
2500	92.18*	91.90*	89.47*	***92.51***	91.96*

Percentages of genotypes with good Hellinger scores (> = 0.45) were analysed. Frameworks showing best performance are written with italic bold letters and the frameworks showing significantly lower performance than the best one are marked with an asterisk (*).

## References

[b1] An P. . Genome-wide association studies identified novel loci for non-high-density lipoprotein cholesterol and its postprandial lipemic response. Human genetics 133, 919–930 (2014). 24604477 10.1007/s00439-014-1435-3 PMC4112746

[b2] van Leeuwen E. M. . Genome of The Netherlands population-specific imputations identify an ABCA6 variant associated with cholesterol levels. Nature communications 6, 6065 (2015). 10.1038/ncomms7065 PMC4366498 25751400

[b3] Zeggini E. . Meta-analysis of genome-wide association data and large-scale replication identifies additional susceptibility loci for type 2 diabetes. Nature genetics 40, 638–645 (2008). 18372903 10.1038/ng.120 PMC2672416

[b4] Lambert J. C. . Meta-analysis of 74,046 individuals identifies 11 new susceptibility loci for Alzheimer’s disease. Nature genetics 45, 1452–1458 (2013). 24162737 10.1038/ng.2802 PMC3896259

[b5] Al Olama A. A. . A meta-analysis of 87,040 individuals identifies 23 new susceptibility loci for prostate cancer. Nature genetics 46, 1103–1109 (2014). 25217961 10.1038/ng.3094 PMC4383163

[b6] Clark A. G. & Li J. Conjuring SNPs to detect associations. Nature genetics 39, 815–816 (2007). 17597769 10.1038/ng0707-815

[b7] Marchini J., Howie B., Myers S., McVean G. & Donnelly P. A new multipoint method for genome-wide association studies by imputation of genotypes. Nature genetics 39, 906–913 (2007). 17572673 10.1038/ng2088

[b8] Peil B., Kabisch M., Fischer C., Hamann U. & Bermejo J. L. Tailored selection of study individuals to be sequenced in order to improve the accuracy of genotype imputation. Genetic epidemiology 39, 114–121 (2015). 25537753 10.1002/gepi.21873

[b9] Abecasis G. R. . An integrated map of genetic variation from 1,092 human genomes. Nature 491, 56–65 (2012). 23128226 10.1038/nature11632 PMC3498066

[b10] Altshuler D. M. . Integrating common and rare genetic variation in diverse human populations. Nature 467, 52–58 (2010). 20811451 10.1038/nature09298 PMC3173859

[b11] International HapMap Consortium. A haplotype map of the human genome. Nature 437, 1299–1320 (2005). 16255080 10.1038/nature04226 PMC1880871

[b12] Frazer K. A. . A second generation human haplotype map of over 3.1 million SNPs. Nature 449, 851–861 (2007). 17943122 10.1038/nature06258 PMC2689609

[b13] Abecasis G. R. . A map of human genome variation from population-scale sequencing. Nature 467, 1061–1073 (2010). 20981092 10.1038/nature09534 PMC3042601

[b14] Burdick J. T., Chen W.-M., Abecasis G. R. & Cheung V. G. In silico method for inferring genotypes in pedigrees. Nature genetics 38, 1002–1004 (2006). 16921375 10.1038/ng1863 PMC3005330

[b15] Li Y., Willer C. J., Ding J., Scheet P. & Abecasis G. R. MaCH: using sequence and genotype data to estimate haplotypes and unobserved genotypes. Genetic epidemiology 34, 816–834 (2010). 21058334 10.1002/gepi.20533 PMC3175618

[b16] Delaneau O. & Marchini J. Integrating sequence and array data to create an improved 1000 Genomes Project haplotype reference panel. Nature communications 5, 3934 (2014). 10.1038/ncomms4934 PMC4338501 25653097

[b17] Howie B., Fuchsberger C., Stephens M., Marchini J. & Abecasis G. R. Fast and accurate genotype imputation in genome-wide association studies through pre-phasing. Nature genetics 44, 955–959 (2012). 22820512 10.1038/ng.2354 PMC3696580

[b18] Liu E. Y., Li M., Wang W. & Li Y. MaCH-admix: genotype imputation for admixed populations. Genetic epidemiology 37, 25–37 (2013). 23074066 10.1002/gepi.21690 PMC3524415

[b19] Shriner D., Adeyemo A., Chen G. & Rotimi C. N. Practical considerations for imputation of untyped markers in admixed populations. Genetic epidemiology 34, 258–265 (2010). 19918757 10.1002/gepi.20457 PMC2912698

[b20] Howie B., Marchini J. & Stephens M. Genotype imputation with thousands of genomes. G3 (Bethesda, Md.) 1, 457–470 (2011). 10.1534/g3.111.001198 PMC3276165 22384356

[b21] Li Y., Willer C., Sanna S. & Abecasis G. Genotype imputation. Annual review of genomics and human genetics 10, 387–406 (2009). 10.1146/annurev.genom.9.081307.164242 PMC2925172 19715440

[b22] Hao K., Chudin E., McElwee J. & Schadt E. E. Accuracy of genome-wide imputation of untyped markers and impacts on statistical power for association studies. BMC genetics 10, 27 (2009). 19531258 10.1186/1471-2156-10-27 PMC2709633

[b23] Huang L. . Genotype-imputation accuracy across worldwide human populations. American journal of human genetics 84, 235–250 (2009). 19215730 10.1016/j.ajhg.2009.01.013 PMC2668016

[b24] Huang L. . Haplotype variation and genotype imputation in African populations. Genetic epidemiology 35, 766–780 (2011). 22125220 10.1002/gepi.20626 PMC3568705

[b25] Jostins L., Morley K. I. & Barrett J. C. Imputation of low-frequency variants using the HapMap3 benefits from large, diverse reference sets. European journal of human genetics: EJHG 19, 662–666 (2011). 21364697 10.1038/ejhg.2011.10 PMC3110048

[b26] Nelson M. R. . The Population Reference Sample, POPRES: a resource for population, disease, and pharmacological genetics research. American journal of human genetics 83, 347–358 (2008). 18760391 10.1016/j.ajhg.2008.08.005 PMC2556436

[b27] dbGaP Homepage. | phs000145.v4.p2 | POPRES: Population Reference Sample. Available at http://www.ncbi.nlm.nih.gov/projects/gap/cgi-bin/study.cgi?study_id=phs000145.v4.p2.

[b28] Loeffler M. . The LIFE-Adult-Study: objectives and design of a population-based cohort study with 10,000 deeply phenotyped adults in Germany. BMC public health 15, 691 (2015). 26197779 10.1186/s12889-015-1983-z PMC4509697

[b29] Roshyara N. R. & Scholz M. fcGENE: a versatile tool for processing and transforming SNP datasets. PloS one 9, e97589 (2014). 25050709 10.1371/journal.pone.0097589 PMC4106754

[b30] Troendle J. F. & Yu K. F. A note on testing the Hardy-Weinberg law across strata. Annals of human genetics 58, 397–402 (1994). 7864594 10.1111/j.1469-1809.1994.tb00735.x

[b31] Homepage of imputation software MaCH1.0. MACH Tutorial - Imputation. Available at http://csg.sph.umich.edu//abecasis/MACH/tour/imputation.html.

[b32] Roshyara N. R. & Scholz M. Impact of genetic similarity on imputation accuracy. BMC genetics 16, 90 (2015). 26193934 10.1186/s12863-015-0248-2 PMC4509609

[b33] Homepage of IMPUTE2. IMPUTE2. Available at https://mathgen.stats.ox.ac.uk/impute/impute_v2.html (2015).

[b34] 1000G PhaseI 2012 v3 Updated Integrated Phase 1 Release. Available at http://csg.sph.umich.edu//abecasis/mach/download/1000G.2012-03-14.html.

[b35] 1,000 Genomes haplotypes – Phase I integrated variant set release (v3) in NCBI build 37 (hg19) coordinates. Available at http://mathgen.stats.ox.ac.uk/impute/data_download_1000G_phase1_integrated.html (2012).

[b36] Delaneau O., Zagury J.-F. & Marchini J. Improved whole-chromosome phasing for disease and population genetic studies. Nature methods 10, 5–6 (2013). 23269371 10.1038/nmeth.2307

[b37] Roshyara N. R., Kirsten H., Horn K., Ahnert P. & Scholz M. Impact of pre-imputation SNP-filtering on genotype imputation results. BMC genetics 15, 88 (2014). 25112433 10.1186/s12863-014-0088-5 PMC4236550

