# Supplementary material for: Comparing performance of modern genotype imputation methods in different ethnicities
Source: Sci Rep. 2016 Oct 4;6:34386. doi: 10.1038/srep34386 (PMC5048136; doi:10.1038/srep34386)
Supplement: Supplementary Information [file srep34386-s1.pdf]

Comparing performance of modern genotype imputation methods in different ethnicities

Nab Raj Roshyara[1,2], Katrin Horn[1], Holger Kirsten[1,2,3], Peter Ahnert[1,2] and Markus Scholz[1,2]

1. Institute for Medical Informatics, Statistics and Epidemiology, University of Leipzig, Haertelstrasse 16-18, 04107 Leipzig, Germany
2. LIFE Center (Leipzig Interdisciplinary Research Cluster of Genetic Factors, Phenotypes and Environment), University of Leipzig, Philipp-Rosenthal Strasse 27, 04103 Leipzig, Germany
3. Department for Cell Therapy, Fraunhofer Institute for Cell Therapy and Immunology, Perlickstraße 1, 04103 Leipzig

Commands used for genotype imputation

MaCH commands for imputation of popres data set with HapMap 3 reference

Step1:

./mach1 -p target_data.ped -d target_data.dat -s hapmap3_ref.snps -h hapmap3_ref.hap.gz --greedy -r 30 --prefix target_data_output_step1

Step2:

./mach1 -p target_data.ped -d target_data.dat -s hapmap3_ref.snps -h hapmap3_ref.hap.gz --crossover target_data_output_step1.rec --errormap target_data_output_step1.rec --greedy --geno --quality --dosage --probs --phase --mle --mldetails --prefix target_data_output_step2

MaCH commands for imputation of LIFE A1 data set with 1000Genomes phase 1 rel. 3 reference

Step1:

./mach1 -p target_data.ped -d target_data.dat --vcfReference -h chr22.phase1_release_v3.20101123.snps_indels_svs.genotypes.refpanel.EUR.vcf.gz --startposition 25553359 --endposition 35553359 --greedy --compact -r 30 --prefix target_data_output_step1

Step2:

./mach1 -p target_data.ped -d target_data.dat --vcfReference -h chr22.phase1_release_v3.20101123.snps_indels_svs.genotypes.refpanel.EUR.vcf.gz --crossover target_data_output_step1.rec --errormap target_data_output_step1.erate --startposition 25553359 --endposition 35553359 --greedy --geno --quality --dosage --probs --phase --mle --mldetails –prefix target_data_output_step2

MaCH-Minimac commands for imputation of popres data set with HapMap 3 reference

Step1:

./mach1 -p target_data.ped -d target_data.dat --rounds 20 --states 200 --phase --interim 5 --sample 5 --prefix target_output_step1

Step2:

./minimac --refSnps hapmap3_ref.snps --refHaps hapmap3_ref.hap --snps snplist_name.txt

--haps target_output_step1.hap.gz --round 20 --states 200 --phased --probs --gzip --em

--prefix target_output_step2

MaCH-Minimac commands for imputation of LIFE A1 data set with 1000Genomes phase 1 rel. 3 reference

Step1:

./mach1 -p target_data.ped -d target_data.dat --rounds 20 --states 200 --phase --interim 5

--prefix target_output_step1

Step2:

./minimac2 --haps target_output_step1.hap.gz --snps snplist_name.txt --vcfReference –refHaps

chr22.phase1_release_v3.20101123.snps_indels_svs.genotypes.refpanel.EUR.vcf.gz --round 20

--states 200 --em --phased --probs --gzip --prefix target_output_step2

MaCH-Admix commands for imputation of popres data set with HapMap 3 reference

./mach-admix -p target_data.ped -d target_data.dat -s reference_data.snp -h reference_data.hap

--geno --probs --dosage --phase --prefix output_data

MaCH-Admix commands for imputation of LIFE A1 data set with 1000Genomes phase 1 rel. 3 reference

./mach-admix -p target_data.ped -d target_data.dat --vcfReference -h

chr22.phase1_release_v3.20101123.snps_indels_svs.genotypes.refpanel.ALL.vcf.gz

--startposition 25553359 --endposition 35553359 --geno --probs --dosage --phase

--prefix output_data

IMPUTE2 commands

./impute2 -m reference_genetic_map.txt -h reference.hap.gz -l reference.legend -g target_data.gens

-strand_g target_data.strand -pgs -int lowerBound upperBound -Ne 20000 -o target_output_name

SHAPEIT-IMPUTE2 commands

Step1:

./shapeit --input-gen target_data.gens target_data.sample --input-map reference_genetic_map.txt --output-max output_data.haps output_data.sample --output-log output_data.log

Step2:

./impute2 -use_prephased_g -known_haps_g output_data.haps -m reference_genetic_map.txt -h reference.hap.gz -l reference.legend -strand_g target_data.strand -iter 50 -burnin 20 -phase -int lowerBound upperBound -Ne 20000 -o target_output_name

A

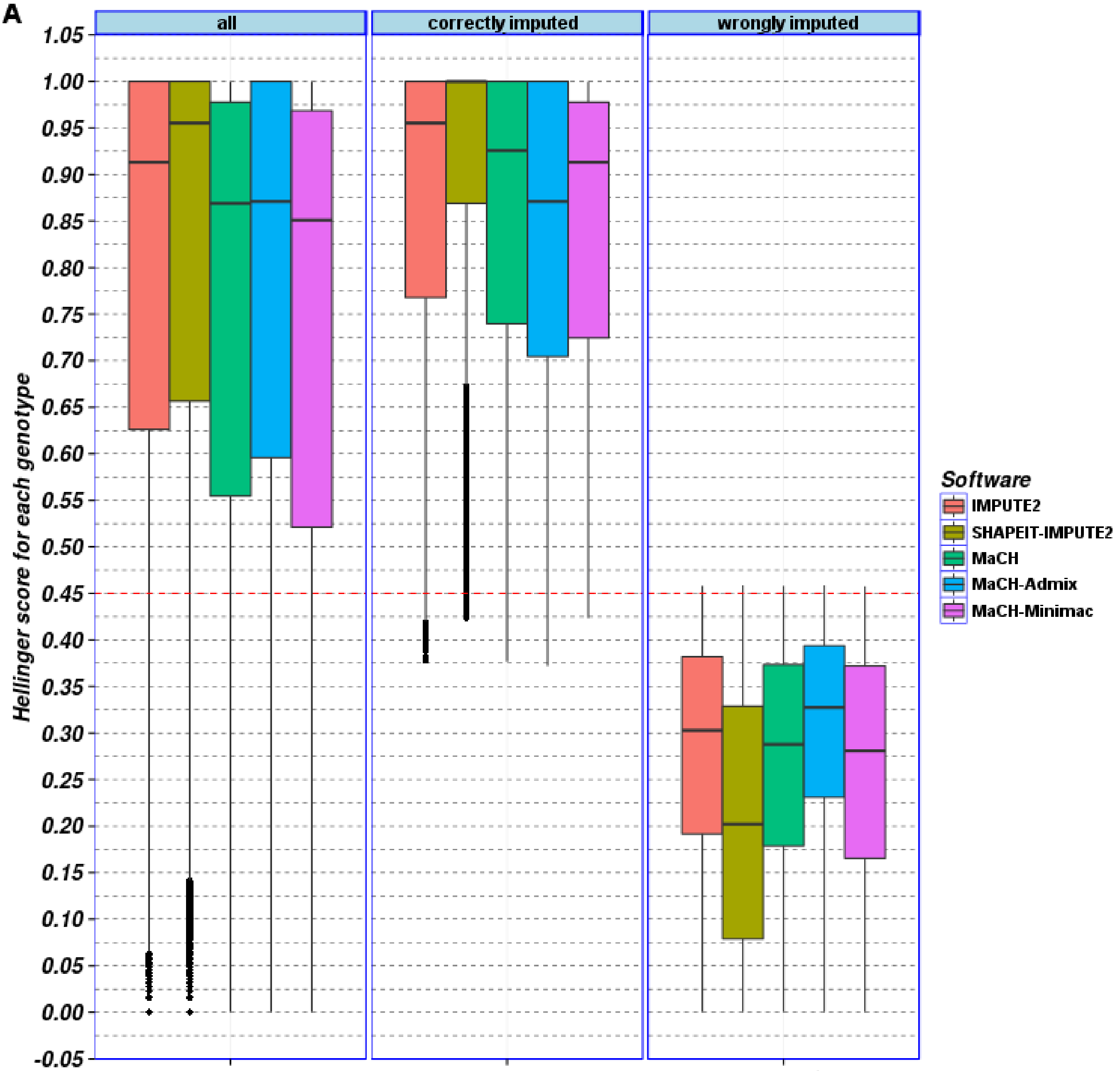

all
correctly imputed
wrongly imputed
1.05
1.00
0.95
0.90
0.85
0.80
0.75
0.70
0.65
0.60
0.55
0.50
0.45
0.40
0.35
0.30
0.25
0.20
0.15
0.10
0.05
0.00
-0.05
Hellinger score for each genotype
Software
IMPUTE2
SHAPEIT-IMPUTE2
MaCH
MaCH-Admix
MaCH-Minimac

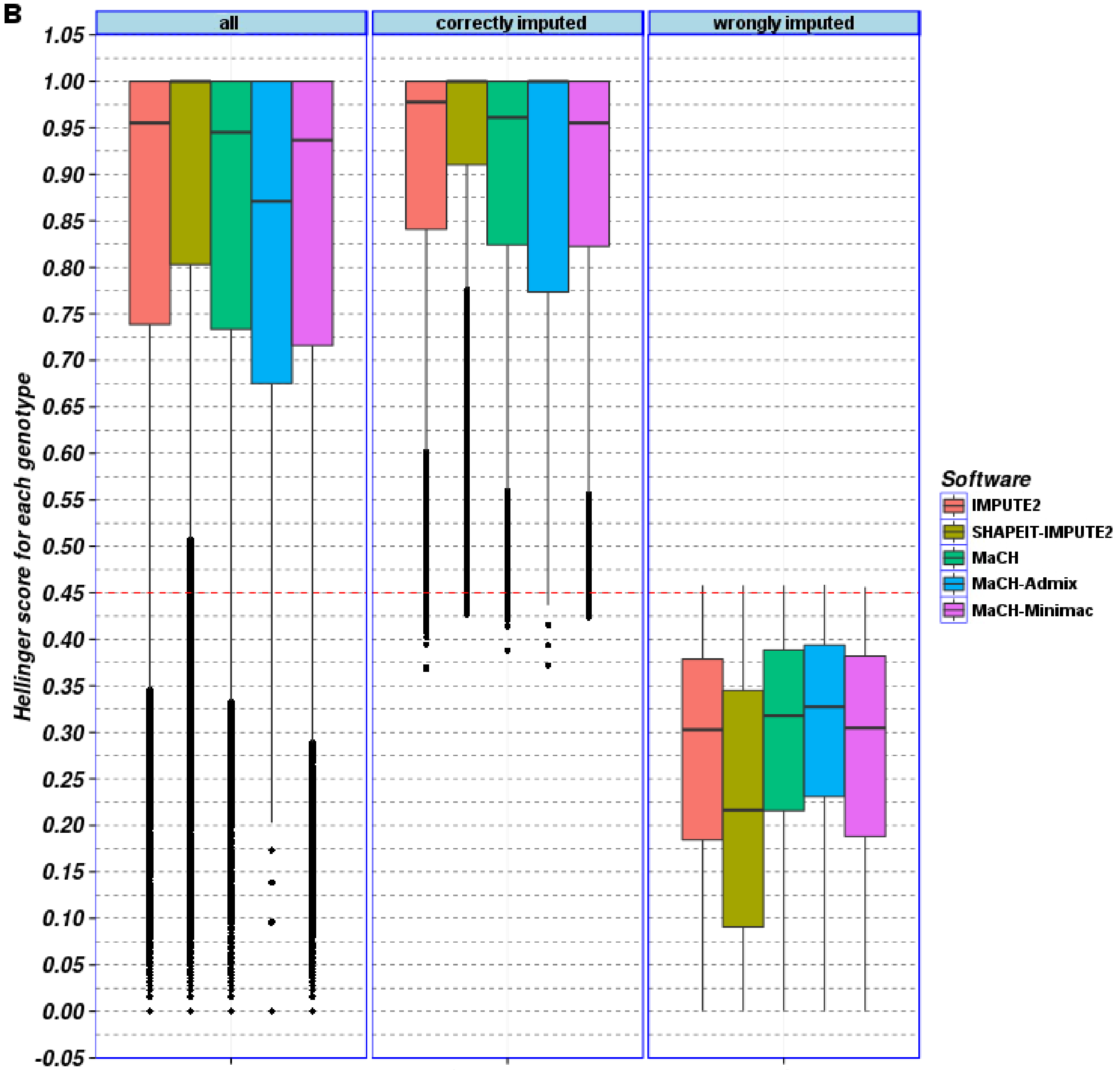
B
all
correctly imputed
wrongly imputed
Hellinger score for each genotype
1.05
1.00
0.95
0.90
0.85
0.80
0.75
0.70
0.65
0.60
0.55
0.50
0.45
0.40
0.35
0.30
0.25
0.20
0.15
0.10
0.05
0.00
-0.05
Software
IMPUTE2
SHAPEIT-IMPUTE2
MaCH
MaCH-Admix
MaCH-Minimac

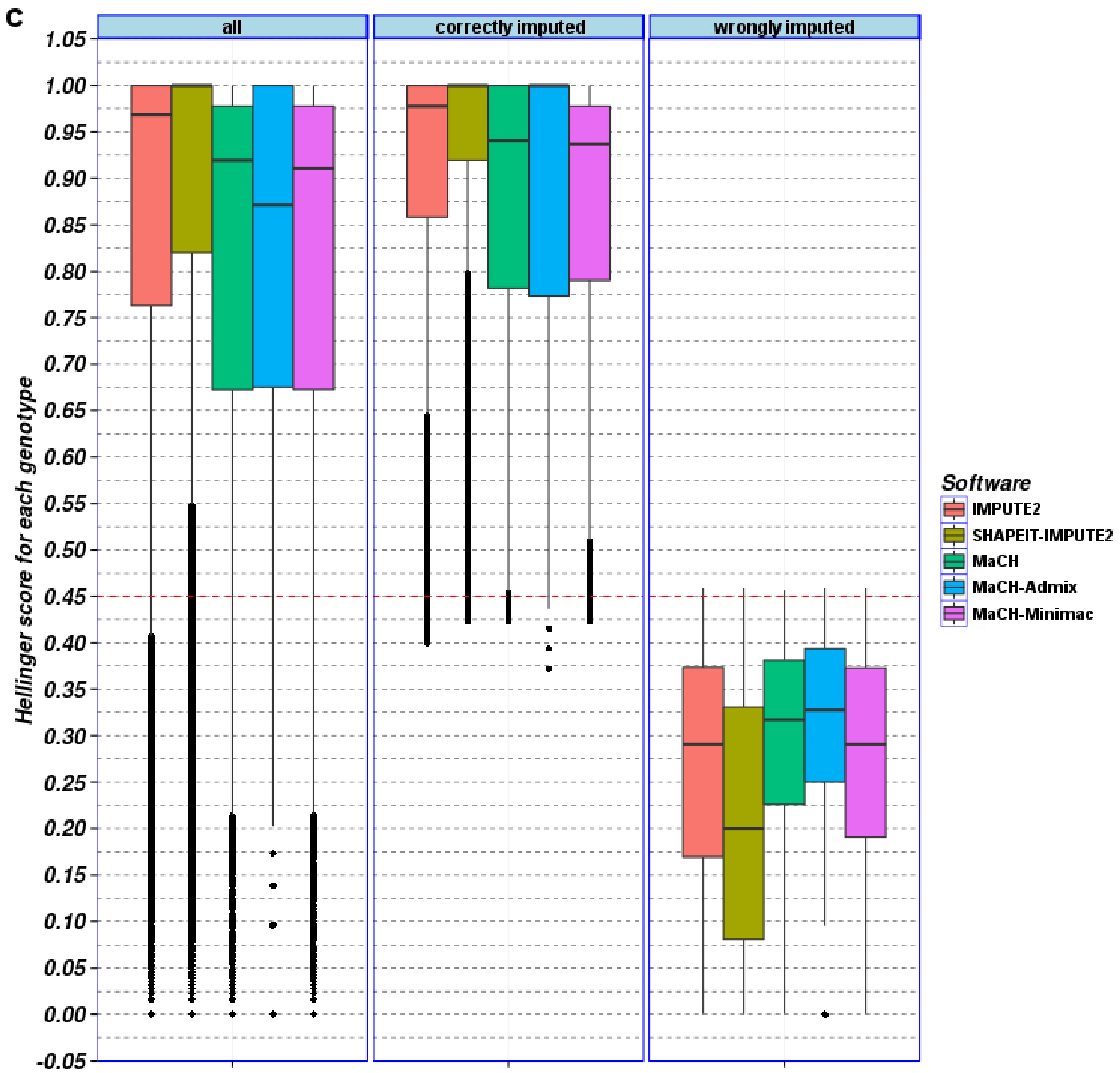

Supplementary figure S1: Boxplots of Hellinger scores of genotypes imputed with five different frameworks. We present results for all imputed genotypes, and separately, for cases where best guess genotypes match true genotypes (correctly imputed) or not (wrongly imputed). A: AfAm, B: Germany, C: Japan POPRES population. As one can see, a Hellinger score >= 0.45 almost ensures that the best-guess genotype equals the true genotype. The figures look very similar for the different POPRES populations considered.

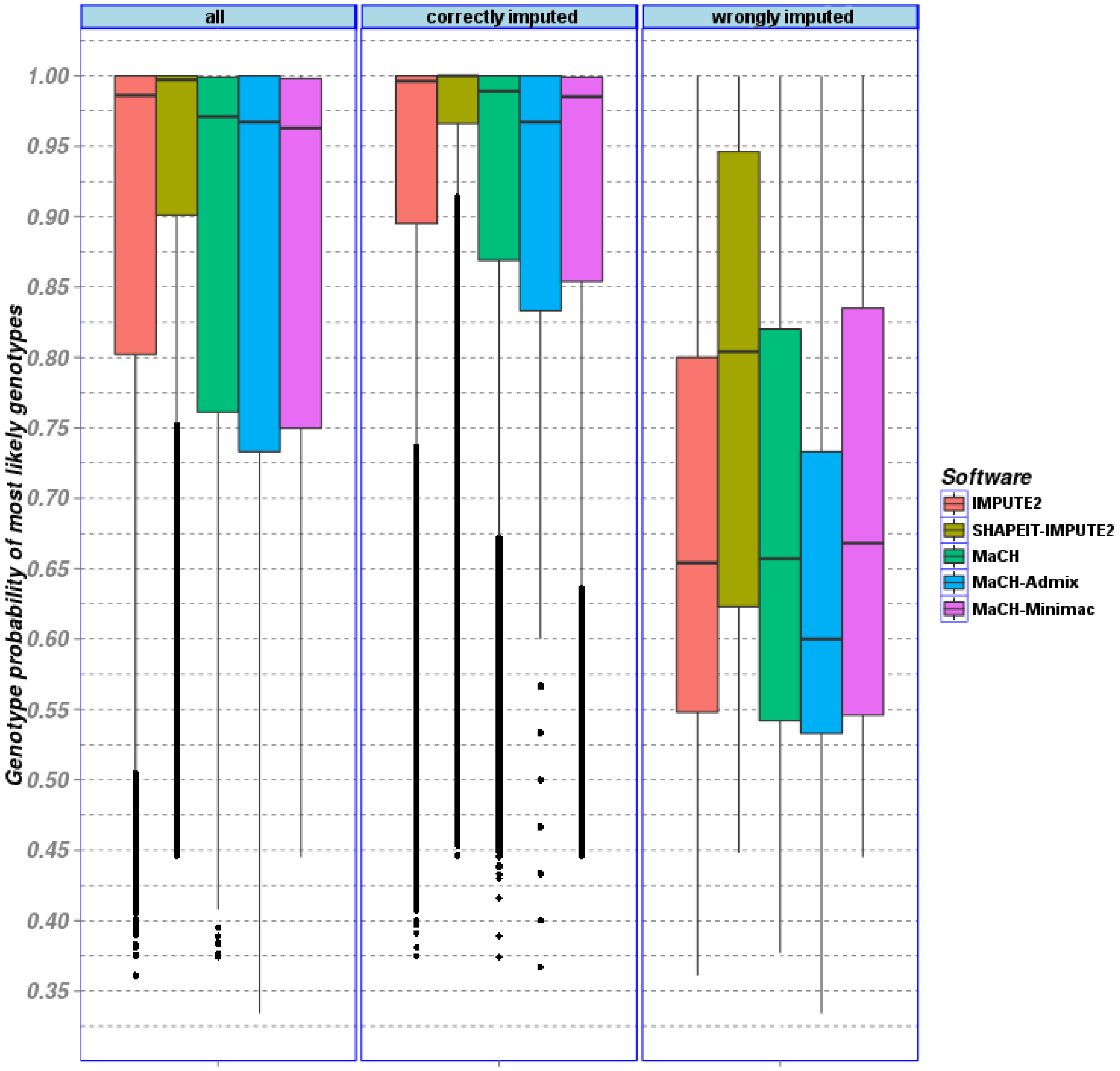

Supplementary figure S2: Boxplots of posterior probabilities of best guess genotypes in AfAm population. All imputation tools were applied with default parameters and reference panels. SHAPEIT-IMPUTE2 shows exceptionally high posterior probabilities for wrongly imputed SNPs.

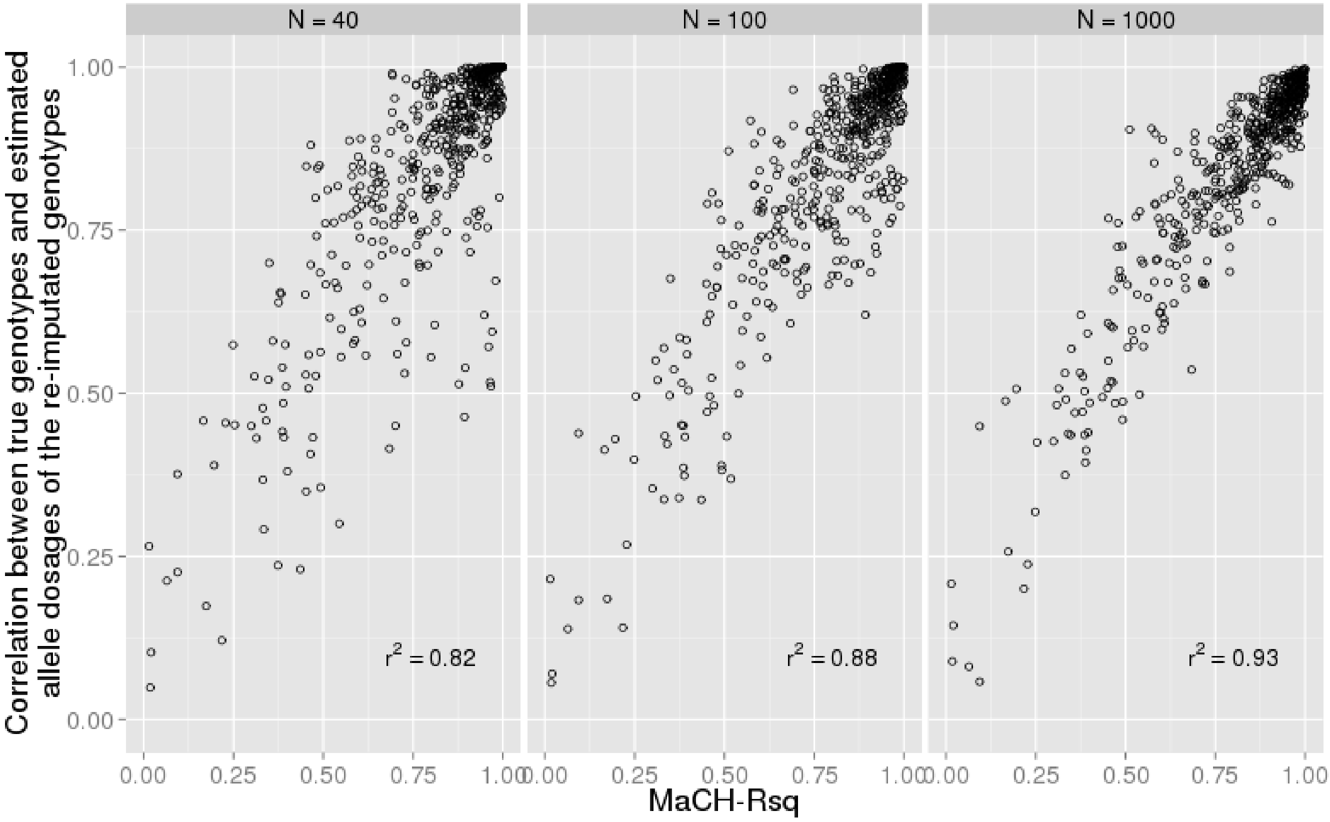

Supplementary figure S3: Comparison of two measures of imputation accuracy for the LIFE-Adult data. Each point represents an imputed HQ-SNP. Analysis was performed in dependence on case numbers of LIFE subsets (N=40, 100, 1000 respectively). As one can see, the two measures are in strong agreement becoming even stronger for larger sample sizes.

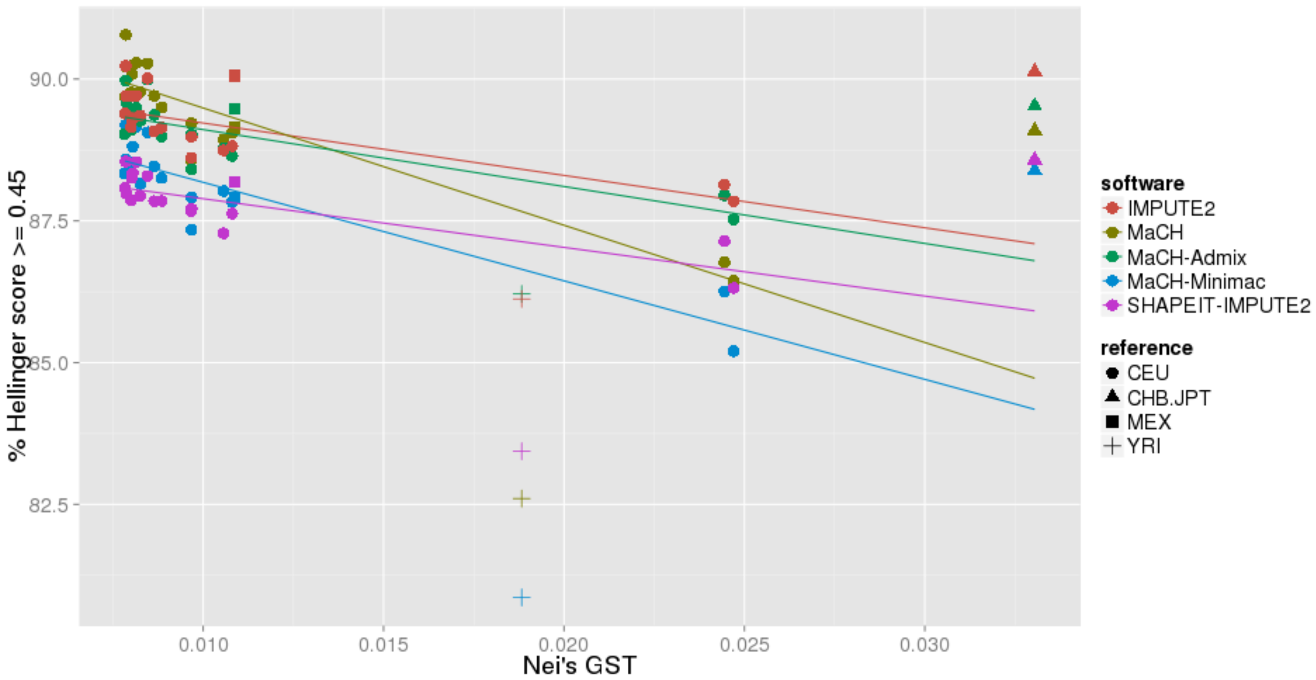

Supplementary Figure S4: Relation between Nei's G_{ST} and imputation accuracy. Symbols correspond to imputation results of POPRES populations. There is a general trend towards lower imputation accuracy for larger distance to the best matching reference. The trend is observed for all imputation frameworks considered. POPRES AfAm population (crosses) results in particularly low imputation quality due to the higher number of polymorphic sites and the weaker linkage structure. POPRES Japanese population (triangles) shows the opposite behavior.

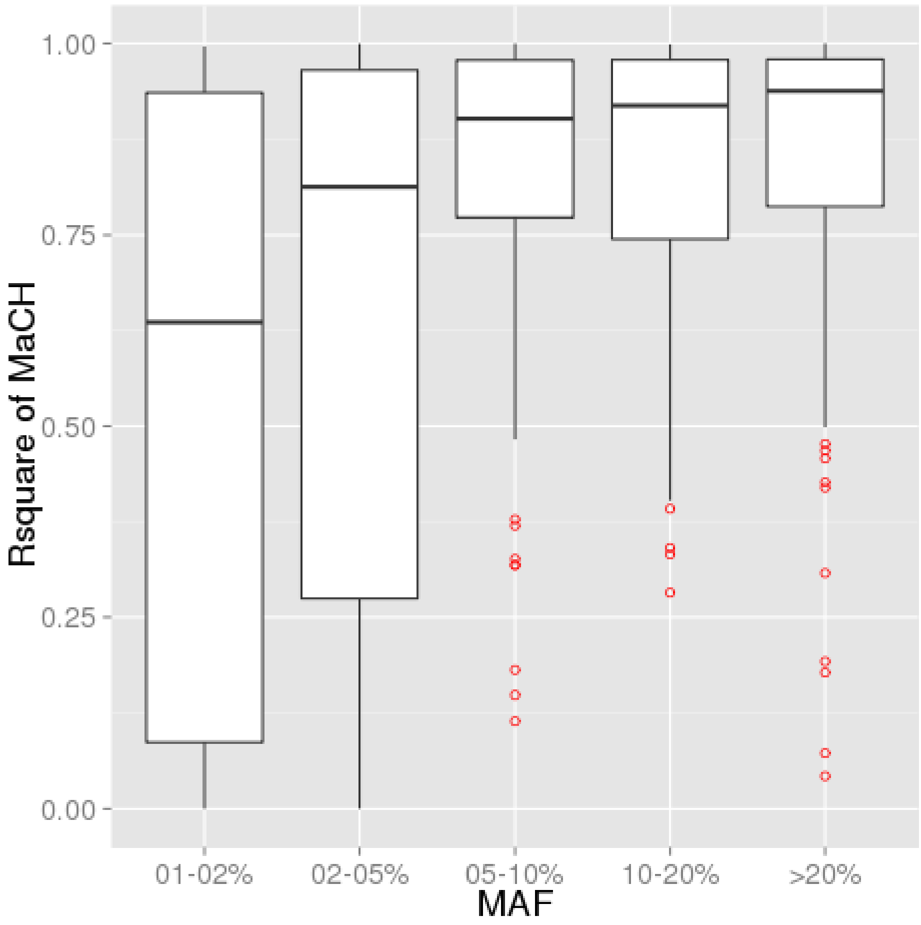

Supplementary Figure S5: Impact of MAF on imputation quality analysed in a subset of N=40 LIFE-Adult samples. SNPs with MAF<5% are particularly prone to inferior imputation quality. Performances of higher frequent variants are comparable.

Country	MaCH and MaCH-Minimac framework (Best-matched Reference Panel)				Mixed Reference Panel		
	Reference Panel	Nei's Gst Score	MaCH Score	MaCH_Minimac Score	MaCH-Admix Score	IMPUTE2 Score	SHAPEIT-IMPUTE2 Score
Australian	CEU	0.0078287	***88.916***	87.691*	88.18*	88.471*	87.329*
British	CEU	0.0078541	***89.962***	88.503*	89.095*	89.392*	87.697*
Canadian	CEU	0.0078631	***89.472***	88.067*	88.632*	88.879*	87.173*
Swiss.French	CEU	0.0079978	***89.027***	88.001*	88.237*	88.264*	87.179*
French	CEU	0.0080226	***89.351***	87.553*	88.244*	88.376*	87.521*
German	CEU	0.0080485	***89.484***	88.169*	88.667*	88.684*	87.423*
Irish	CEU	0.0081449	***89.474***	88.255*	88.771*	88.788*	87.767*
Swiss	CEU	0.0082549	***89.100***	87.515*	88.316*	88.65*	87.137*
Belgians	CEU	0.0084603	***89.354***	88.143*	88.935*	89.078	87.763*
Swiss.German	CEU	0.0086417	***88.813***	87.415*	88.402*	88.106*	86.966*
eastEU	CEU	0.0088483	***88.656***	87.462*	88.114*	88.349	87.111*
Portuguese	CEU	0.0096742	***87.642***	86.627*	87.554	87.554	86.879*
Spanish	CEU	0.0096786	88.337	87.01*	***88.409***	88.079	87.097*
Italian	CEU	0.0105699	***87.822***	87.017*	87.822	87.652	86.513*
From Yugoslavia	CEU	0.0108079	***88.276***	87.015*	87.76*	87.623*	86.702*
Mexican	MEX	0.0108799	88.347*	87.501*	88.775*	***89.192***	87.348*
AfAm	YRI	0.0188273	81.655*	79.961*	***85.197***	85.092	82.526*
Punjabi	CEU	0.0244462	85.873*	85.67*	***87.271***	87.194	86.295*
Indian	CEU	0.0247062	85.783*	84.714*	***87.105***	87.044	85.663*
Japanese	CHB.JPT	0.0330444	88.525*	87.978*	88.558*	***89.368***	87.822*

Supplementary Table S1: Comparison of percentages of genotypes with good SEN scores (>=0.95) obtained for 20 different POPRES samples with either MaCH, MaCH-Minimac, MaCH-Admix, IMPUTE2, or SHAPEIT-IMPUTE2. For Imputation with MaCH and MaCH-Minimac framework, the best matched reference panel based on Nei's G_{ST} was used. Nei's G_{ST} values and corresponding reference panels are also presented. Imputation frameworks with best results are marked with bold italic letters for each population. Scenarios significantly inferior to the best one are marked with an asterisk. McNemar's test was applied for this purpose.

	MaCH and MaCH-Minimac framework (Best-matched Reference Panel)				Mixed Reference Panel		
**Country**	**Reference Panel**	**Nei's Gst Score**	**MaCH Score**	**MaCH-Minimac Score**	**MaCH-Admix Score**	**IMPUTE2 Score**	**SHAPEIT-IMPUTE2 Score**
Australian	CEU	0.0078287	***90.579***	89.273*	89.876*	89.882*	88.197*
British	CEU	0.0078541	***91.536***	90.001*	90.862*	90.697*	88.58*
Canadian	CEU	0.0078631	***91.019***	89.4*	90.777	90.251*	88.073*
Swiss.French	CEU	0.0079978	***90.513***	89.279*	89.882*	89.498*	88.067*
French	CEU	0.0080226	***91.012***	89.291*	90.206*	89.718*	88.359*
German	CEU	0.0080485	***91.002***	89.714*	90.53*	89.999*	88.377*
Irish	CEU	0.0081449	***91.071***	89.957*	90.588*	90.176*	88.656*
Swiss	CEU	0.0082549	***90.613***	88.979*	90.213	89.851*	88.069*
Belgians	CEU	0.0084603	90.978	89.794*	***90.983***	90.416*	88.44*
Swiss.German	CEU	0.0086417	***90.430***	89.131*	90.216	89.498*	88.018*
eastEU	CEU	0.0088483	***90.185***	89.106*	90.010	89.5*	87.927*
Portuguese	CEU	0.0096742	89.255	88.262*	***89.430***	89.008*	87.889*
Spanish	CEU	0.0096786	89.983	88.754*	***90.213***	89.5*	87.865*
Italian	CEU	0.0105699	89.734	88.841*	***89.783***	89.235*	87.449*
From Yugoslavia	CEU	0.0108079	***89.713***	88.578*	89.685	89.186*	87.673*
Mexican	MEX	0.0108799	89.763*	88.808*	90.433	***90.454***	88.248*
AfAm	YRI	0.0188273	83.535*	81.901*	***87.231***	86.677*	83.574*
Punjabi	CEU	0.0244462	87.6*	87.107*	***89.146***	88.669*	87.288*
Indian	CEU	0.0247062	87.247*	86.244*	***88.629***	88.415*	86.501*
Japanese	CHB.JPT	0.0330444	90.183	89.401*	90.501	***90.529***	88.659*

Supplementary Table S2: Counts (in percentage) of most likely genotypes which are well-matched with the original genotypes as obtained for 20 different POPRES samples with either MaCH, MaCH-Minimac, MaCH-Admix, IMPUTE2, or SHAPEIT-IMPUTE2. For Imputation with MaCH and MaCH-Minimac framework, the best matched reference panel based on Nei's G_{ST} was used. Nei's G_{ST} values and corresponding reference panels are also presented. Imputation frameworks with best results are marked with bold italic letter for each population. Scenarios significantly inferior to the best one are marked with an asterisk. McNemar's test was applied for this purpose.

	MaCH and MaCH-Minimac framework (Best-matched Reference Panel)				Mixed Reference Panel		
**Country**	**Reference Panel**	**Nei's Gst Score**	**MaCH Score**	**MaCH-Minimac Score**	**MaCH-Admix Score**	**IMPUTE2 Score**	**SHAPEIT-IMPUTE2 Score**
Australian	CEU	0.0078287	0.808*	***0.815***	0.767*	0.829*	***0.878***
British	CEU	0.0078541	0.814*	***0.817***	0.773*	0.834*	***0.879***
Canadian	CEU	0.0078631	0.811*	***0.817***	0.768*	0.832*	***0.879***
Swiss.French	CEU	0.0079978	0.809*	***0.819***	0.772*	0.834*	***0.880***
French	CEU	0.0080226	0.812*	***0.816***	0.773*	0.834*	***0.880***
German	CEU	0.0080485	0.812*	***0.820***	0.771*	0.833*	***0.878***
Irish	CEU	0.0081449	0.813*	***0.819***	0.769*	0.835*	***0.879***
Swiss	CEU	0.0082549	0.811*	***0.813***	0.77*	0.834*	***0.879***
Belgians	CEU	0.0084603	0.813*	***0.815***	0.774*	0.835*	***0.878***
Swiss.German	CEU	0.0086417	0.809*	***0.814***	0.769*	0.831*	***0.878***
eastEU	CEU	0.0088483	0.806*	***0.815***	0.766*	0.831*	***0.877***
Portuguese	CEU	0.0096742	0.802*	***0.808***	0.762*	0.825*	***0.875***
Spanish	CEU	0.0096786	0.802*	***0.811***	0.762*	0.825*	***0.875***
Italian	CEU	0.0105699	0.803*	***0.808***	0.762*	0.821*	***0.872***
From Yugoslavia	CEU	0.0108079	0.806*	***0.813***	0.765*	0.829*	***0.879***
Mexican	MEX	0.0108799	0.797*	***0.799***	0.77*	0.838*	***0.879***
AfAm	YRI	0.0188273	0.716*	0.712	***0.719***	0.777*	***0.842***
Punjabi	CEU	0.0244462	0.788*	***0.800***	0.756*	0.815*	***0.870***
Indian	CEU	0.0247062	0.789*	***0.796***	0.761*	0.82*	***0.868***
Japanese	CHB.JPT	0.0330444	0.759*	***0.778***	0.756*	0.832*	***0.876***

Supplementary Table S3: Comparison of software specific Rsq score and Info score as obtained for 20 different POPRES samples with either MaCH, MaCH-Minimac, MaCH-Admix, IMPUTE2, or SHAPEIT-IMPUTE2. For Imputation with MaCH and MaCH-Minimac framework, the best matched reference panel based on Nei's G_{ST} was used. Nei's G_{ST} values and corresponding reference panels are also presented. Imputation frameworks with best results are marked with bold italic letters for each population. Scenarios significantly inferior to the best one are marked with an asterisk. McNemar's test was applied for this purpose.

Country	Genetic similarity		MaCH-Minimac			SHAPEIT-IMPUTE2		
	Reference Panel	Nei_Gst	50%	70%	100%	50%	70%	100%
Australian	CEU	0.0078287	***90.168***	***89.093***	***88.414***	87.877*	88.502	88.041
British	CEU	0.00785414	***90.451***	***89.51***	***89.226***	88.657*	88.733*	88.285*
Canadian	CEU	0.00786305	***90.404***	***89.124***	***88.894***	88.314*	88.084*	87.8*
Swiss.French	CEU	0.00799776	***89.64***	***88.809***	***88.688***	88.185*	88.043*	88.141
French	CEU	0.00802259	***89.714***	***89.244***	88.128	88.303*	88.018*	***88.325***
German	CEU	0.00804851	***90.226***	***89.341***	***88.882***	88.368*	88.499*	88.018*
Irish	CEU	0.00814486	***89.562***	***88.949***	***88.554***	87.919*	87.842*	88.05
Swiss	CEU	0.00825494	***89.819***	***88.801***	***88.32***	88.057*	87.98*	87.98
Belgians	CEU	0.00846027	***90.086***	***89.36***	***88.789***	88.459*	88.151*	88.338
Swiss.German	CEU	0.00864172	***89.623***	***88.496***	***87.851***	87.305*	87.622*	87.436
eastEU	CEU	0.00884828	***89.359***	***88.364***	***88.134***	87.369*	87.62*	87.762
Portuguese	CEU	0.00967424	***88.734***	***87.661***	87.136	87.005*	87.037	***87.18***
Spanish	CEU	0.00967859	***89.08***	***88.161***	***87.712***	87.395*	87.11*	87.635
Italian	CEU	0.0105699	***88.75***	***87.865***	***87.996***	87.231*	87.329	87.198*
From Yugoslavia	CEU	0.0108079	***89.102***	***88.303***	***88.029***	87.624*	87.329*	87.285*
Mexican	MEX	0.0108799	***89.571***	***88.99***	***88.563***	88.727*	88.782	88.42
AfAm	YRI	0.0188273	82.212	80.91*	80.659*	***82.628***	***82.376***	***82.89***
Punjabi	CEU	0.0244462	***87.693***	86.938	86.577*	87.036	***87.233***	***87.244***
Indian	CEU	0.0247062	***87.137***	86.36	85.66*	86.7	***86.71***	***86.7***
Japanese	CHB.JPT	0.0330444	***89.583***	88.843	88.977	89.101	***89.078***	***89.033***

Supplementary Table S4: Percentage of genotypes with good SEN score (>=0.95) for imputation frameworks with pre-phasing strategy. Different percentages of HQ-SNPs were masked (50%, 70%, 100%). Imputation frameworks with best results are marked with bold italic letters for each population. Scenarios significantly inferior to the best one are marked with an asterisk. McNemar's test was applied for this purpose.
